# Verification of a Rapidly Multiplexed Circuit for Scalable Action Potential Recording

**DOI:** 10.1109/TBCAS.2019.2958348

**Published:** 2019-12-09

**Authors:** Mohit Sharma, Hunter Joseph Strathman, Ross Martin Walker

**Affiliations:** Department of Electrical and Computer Engineering, University of Utah, Salt Lake City, UT 84112 USA; Department of Biomedical Engineering, University of Utah, Salt Lake City, UT 84112 USA; Department of Electrical and Computer Engineering, University of Utah, Salt Lake City, UT 84112 USA

**Keywords:** Action potential, analog design, electrode, electrode array, neural amplifier, neural engineering, neural recording, neuroscience, windowed integrator sampling

## Abstract

This report presents characterizations of *in vivo* neural recordings performed with a CMOS multichannel neural recording chip that uses rapid multiplexing directly at the electrodes, without any pre-amplification or buffering. Neural recordings were taken from a 16-channel microwire array implanted in rodent cortex, with comparison to a gold-standard commercial bench-top recording system. We were able to record well-isolated threshold crossings from 10 multiplexed electrodes and typical local field potential waveforms from 16, with strong agreement with the standard system (average SNR = 2.59 and 3.07 respectively). For 10 electrodes, the circuit achieves an effective area per channel of 0.0077 mm^2^, which is >5x smaller than typical multichannel chips. Extensive characterizations of noise and signal quality are presented and compared to fundamental theory, as well as results from *in vivo* and *in vitro* experiments. By demonstrating the validation of rapid multiplexing directly at the electrodes, this report confirms it as a promising approach for reducing circuit area in massively-multichannel neural recording systems, which is crucial for scaling recording site density and achieving large-scale sensing of brain activity with high spatiotemporal resolution.

## I. Introduction

ELECTROPHYSIOLOGICAL recording is the gold standard for measuring neural activity due to its potential for high spatiotemporal resolution [1]. Extracellular electrical recordings are typically made with multichannel electrode arrays implanted in brain tissue. The number of recorded neurons has steadily increased as electrode technology has improved over time [2]. However, the number of available neurons is still many orders of magnitude smaller than the total number of neurons in the brain. Therefore, large efforts are underway to increase the number of implanted electrodes, in order to provide a more complete picture of neural activity. Through large-scale recording, researchers wish to decode circuit activity in “cell assemblies,” and also understand large functional networks in the brain [1].

Extracellular recordings consist of low frequency local field potentials (LFPs) with signal content up to 300 Hz and amplitudes up to 3 mV [3], and action potentials (APs) with useful signal bandwidth between 300 Hz to 10 kHz and amplitudes ranging from 10 *μ*V to 1 mV [4]. Neural recording integrated circuits (ICs) are usually designed for 500–1000 V/V gain, 2–7 *μ*V_rms_ total integrated noise, and 5–10 kHz bandwidth, typically resulting in ~10 *μ*W/channel power dissipation and ~0.04 mm^2^/channel area [5].

These CMOS front-end ICs traditionally consist of a dedicated amplifier for each electrode, a lowpass anti-aliasing filter up to 10 kHz, and analog to digital converters (ADCs) that may or may not be multiplexed across channels. However, it is challenging to scale this architecture to higher than a few hundred electrodes, due to large area [5]. Flicker noise in the signal band and low frequency filtering typically result in high area utilization from transistors and capacitors, respectively. To address circuit area, we have investigated sharing a single front-end circuit among multiple channels via rapid time domain multiplexing, directly at the electrodes, without any preamplification or buffering [6]. This technique divides the area per channel by the number of multiplexed electrodes (the multiplexing factor). However, the technique also presents a number of challenges related to noise aliasing and electrode offsets that are not easily solved using traditional approaches.

After summarizing the design, which was described in detail in [6], this report presents verification of experimental results for a rapidly multiplexed CMOS front-end chip implemented in 180 nm CMOS. This verification is important because the 2-electrode multiplexing demonstration in [6] does not establish the feasibility of multiplexing many electrodes in general. To demonstrate the efficacy of this new technique, neural recordings from a microwire array implanted in rodent cortex were characterized for signal quality and noise, and compared to a commercial recording system. Thus, successful recordings from 10 multiplexed electrodes are demonstrated, and the noise theory presented in [6] is quantitatively validated.

The paper is organized as follows. Section II gives a brief description of the circuit architecture and operation. Section III describes the experimental methods. Section IV presents characterizations of recordings from *in vivo* experiments with comparison to the standard bench-top system. Section V presents additional experiments to confirm the noise performance and theory. Section VI discusses the results, application scenarios, and offers avenues for increasing the multiplexing factor. Conclusions are presented in Section VII.

## II. Circuit Architecture

**Fig. 1.**
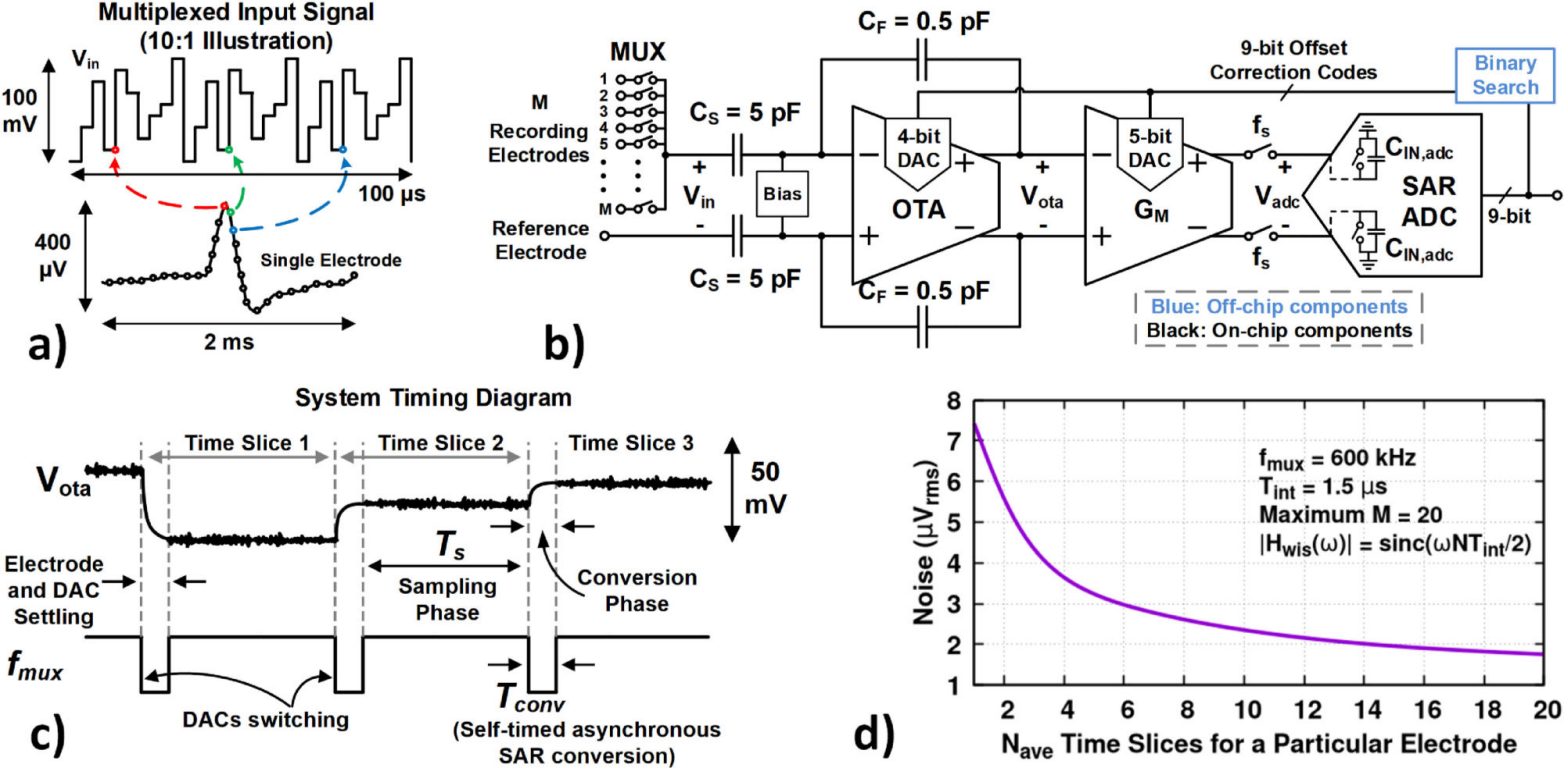
(a) Illustration of the concept of rapid time domain multiplexing for action potential recording. M total electrodes are rapidly switched to a single recording circuit. The overall input signal is dominated by modulated electrode offsets. (b) Overview of the CMOS test chip architecture, which was described in [6]. The effective area per channel of the circuit is divided by M. (c) Timing diagram showing the circuit operation. (d) Projection of the expected total integrated noise from the electrode-tissue interface as a function of the number of timing slices (N_ave_) assigned to a particular electrode. The calculation uses *in vivo* thermal noise data from our experiments [9], and the WIS transfer function shown in the figure to calculate noise across all frequencies (spike filtering will reduce this noise).

The rapidly multiplexed circuit architecture is shown in Fig. 1, and was described in detail in [6]. Multiple electrodes are sequentially switched to the input at a high rate to provide nearly simultaneous sampling at traditional recording rates (~30 kS/s/channel). This architecture departs from traditional neural recording systems due to the higher bandwidth that is required for signal settling. The higher bandwidth also implies that wide-band noise from the electronics and the bio-electrochemical system formed by the electrodes and tissue cannot simply be lowpass filtered, which threatens aliasing into the signal band. Additionally, the DC offsets of the multiplexed electrodes appear as voltage steps at the input. These fast transients cannot simply be highpass filtered, because of the short acquisition time per channel (T_int_ = 1.5 *μs* in this report). Appropriate design choices were made to mitigate these challenges. Windowed integration sampling (WIS) was employed to reduce aliased noise, resulting in smaller noise-equivalent bandwidth (NEB) than traditional voltage sampling (3.5x smaller compared to 0.1% voltage settling) [6]-[8]. Two stages of DACs are used to remove the electrode offset voltages from the signal path, to allow high overall gain before the ADC.

The fully differential signal chain consists of a capacitive feedback preamplifier, followed by a WIS circuit composed of a transconductance amplifier (G_m_) that drives current onto the input capacitance of a successive approximation register (SAR) ADC. One input of the preamplifier is connected to a 32-channel multiplexer constructed from NMOS switches, and the other input is connected to a typically low impedance reference electrode (a cranial bone screw in these experiments).

The number of multiplexed electrodes is the multiplexing factor, M. The maximum number of available time slices that can be assigned to different electrodes is given by N_tot_ = f_mux_/fs, where f_mux_ is the multiplexer base clock frequency and f_s_ is the desired sampling rate per channel. These slices can be uniformly distributed across the electrodes, or alternatively, multiple samples (N_ave_) can be averaged to reduce noise from the circuits and the electrode-tissue interface. In the experiments described in this report, f_mux_ was fixed at 600 kHz and N_tot_ = 20, allowing f_s_ = 30 kS/s/channel for a maximum M of 20 (at N_ave_ = 1). Hence, the multiplexer runs at f_mux_ = 600 kHz, corresponding to 1.67 *μs* per time slice. Of this, the initial 10% was set aside for settling of the preamplifier and DACs, leaving T_int_ = 1.5 *μs* for WIS, which results in a NEB of 333 kHz (see [6] for theory).

**Fig. 2.**
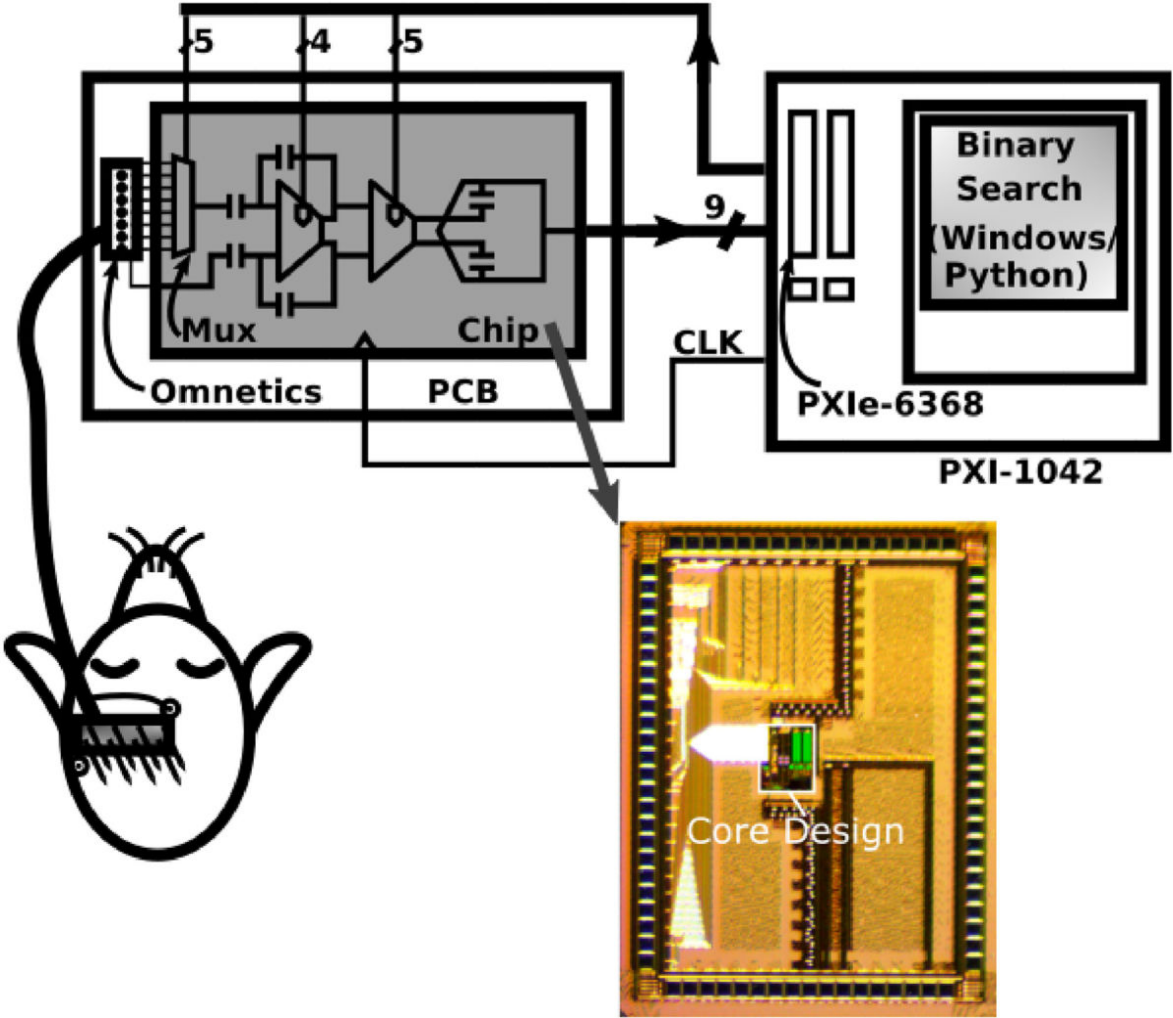
Test setup used for multiplexed neural recordings from an anesthetized rat. The implanted electrode array connects to the multiplexing test chip through a wired Omnetics connector. The digital outputs from the chip are read by a NI PXIe-6368, which also controls the multiplexer addresses, the offset DAC codes, and the master clock. The PXIe-6368 is controlled by a Python script, which implements the binary search algorithm for finding the DAC codes for offset compensation.

As each electrode is selected, the corresponding DAC codes are updated to cancel the electrode offset. The DAC codes are computed by a binary search algorithm, which tries to center the ADC output codes. The electrode offsets drift with time [9], and hence the offset correction DACs are updated periodically. We observed drift rates of approximately 300 *μ*V/s across channels. Therefore, the offset DACs were recalibrated every 3.5 seconds. The binary search was implemented off-chip for flexibility during testing, and takes ~10 ms in the Windows/Python environment (see Figs. 1 and 2). The offset correction range is ±65 mV, split into 4-bit coarse correction in the preamplifier and 5-bit fine correction in the G_m_ amplifier, leading to an input referred resolution of ±250 *μ*V.

The 180 nm CMOS test chip achieves a noise-efficiency factor of 4.74 (regardless of M or N_ave_) and an area of 0.0077 mm^2^/channel for M = 10, which is >5x smaller than typical multichannel chips [5], and reaches the state of the art [10].

## III. Methods

### A. Surgical Procedures

One 16-channel tungsten microwire array (Tucker-Davis Technologies, TDT) was implanted in the cortex of a 400 g, male Sprague Dawley rat. The 50 *μm* diameter electrodes were arranged in two rows of eight with 250 *μm* and 500 *μm* spacing between row and column sites, respectively. In each row, the electrodes ranged from 1 to 3.8 mm in length to follow the curvature of the brain, so that each electrode tip was at approximately the same cortical depth. Tips were cut at a 60° angle to prevent depression of brain tissue during insertion. All studies were conducted with the approval of the Institutional Animal Care and Use Committee at the University of Utah

For implantation, the animal was anesthetized using 3–5% isoflurane for induction, and maintained at 1–2%. The animal was fixed in a stereotactic frame and a rectangular incision was made to expose the skull. A medial to lateral line was drawn 3.8 mm posterior to the bregma, and a hand drill was used to create an opening approximately 2 mm × 4 mm, as close to the temporal ridge as possible. Four small pilot holes were drilled along both temporal ridges and filled with bone screws. Two of these screws were used as ground and reference, the other two were for mechanical support. The array was then inserted into the cortex using a micromanipulator. During insertion, neural activity was monitored until a depth was reached that provided frequent spiking activity across multiple channels. The exposed cortex was then covered with Kwik-Cast silicone elastomer (World Precision Instruments) and UV cure epoxy.

### B. Data Acquisition

The reported recording sessions occurred 5 days after surgery. All neural activity was recorded using either a commercial Grapevine Neural Interface System (Ripple Neuro) referred to as “Grapevine,” or the custom test chip (specifications are shown in Table I). All side-by-side comparison data were taken from the same recording session; however, these recordings were not made simultaneously. This is important to note, since exact waveform matching should not be expected. For Grapevine recordings, signals were first passed through a front end (Micro2+Stim, Ripple) with 1 Hz highpass and 4.9 kHz lowpass filters. The front-end achieves <2.1 *μ*V_rms_ input referred noise, and can be considered a “gold standard” reference (custom neural recording chips generally have significantly higher noise [5]). Signals were digitized at 30 kS/s by an onboard 16-bit ADC, then passed to a multichannel processor (Grapevine Neural Interface Processor, Ripple) before being transmitted to a computer via ethernet for storage and analysis. Recordings were monitored and saved using the Trellis Software Suite (Ripple).

The setup for recordings with the multiplexed chip consisted of a custom test PCB for reference voltage generation and power management, and a National Instruments PXIe 6368 for digital control, clocking and I/O. The implanted array was connected to the test PCB with an Omnetics Nano Strip connector. The 6368 was controlled using custom Python code, and provided the 600 kHz multiplexer clock, addresses, and DAC codes. For most recordings, a repeated sequence of 20 addresses were used to distribute time slices across the available channels, using f_mux_ = 600 kHz and f_s_ = 30 kS/s. For instance, for M = 4 and when using a uniform distribution, each channel would be sampled 5 times (N_ave_ = 5 slices) in one sequence. The N_ave_ slices are averaged to reduce noise. Other variants included 16 slice repetitions and f_s_ = 37.5 kS/s.

All experiments were performed under anesthesia, using a ke- tamine/xylazine cocktail (50 and 7 mg/kg, respectively), which was injected into the intraperitoneal space. Besides recording experiments, electrode characterization measurements were made with the animal inside of a custom copper mesh Faraday cage, and consisted of electrical impedance spectroscopy (EIS) in a 3-electrode configuration using a Gamry Interface 1000E Potentiostat, similarly to [11].

### C. Signal Processing

The recorded data were demultiplexed to separate 30 kS/s streams, which involved averaging samples from multiple time slices together for electrodes that were allocated N_ave_ > 1 (to reduce noise). The data were then processed by digital filters in MATLAB that approximate Grapevine’s analog filters, which is important for comparing waveform shapes since the custom chip’s frequency response is nearly flat [6]. These “raw” data were passed through a digital bandpass “spike filter” consisting of a 4th order highpass (750 Hz) and 4th order lowpass (4000 Hz) zero-phase digital Butterworth filter, in MATLAB. Putative action potentials were isolated using custom MATLAB detection software. Briefly, a threshold was placed at — 3.0x the standard deviation of the spike filtered recordings. Around each threshold crossing, a 1.6 ms waveform snippet was extracted. Isolation of threshold crossing events was performed with time-amplitude window thresholds similar to the “hoops” described in [12]. Upper and lower “hoop” bounds were +/− 1.7x the standard deviation of all the threshold crossing events for the electrode, from a template waveform (Grapevine: mean of threshold crossing events; test chip: mean of sorted events of the same electrode from the Grapevine recordings).

## IV. Signal Analysis and Comparisons

### A. Recorded Waveforms Over Long Timescales

**Fig. 3.**
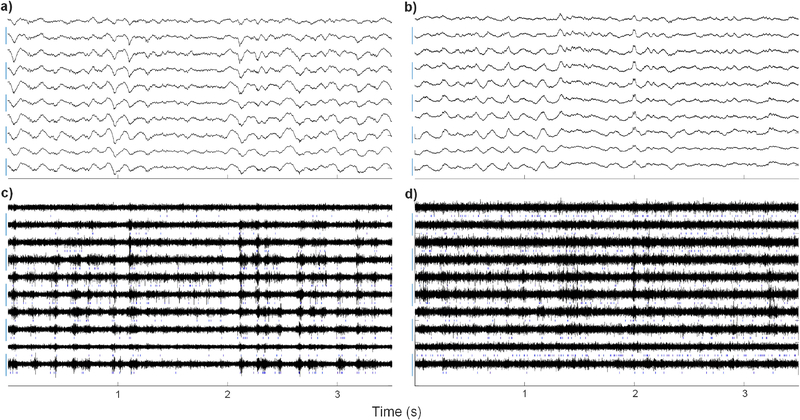
Long time-scale recordings (3.5 sec) from 10 electrodes taken with the Grapevine system (left column) and custom CMOS test chip (right column). The upper figures show “raw” data (see Section III) with an additional 4.9 kHz digital lowpass filter. The lower figures show the spike filtered data (see Section III) for both systems, generated from the same time segments as the upper waveforms. Scale bars on the y axes show 1000 *μ*V (a, b) and 50 *μ*V (c, d). Blue tics indicate locations of threshold crossing events.

Fig. 3 shows 3.5 sec input-referred recordings, where the raw data were either lowpass filtered (top) or spike filtered (bottom). Spike waveforms from the test chip were obtained using M = 10 (2 slices per channel). Grapevine recordings were performed approximately 10 minutes before the multiplexed recordings in an effort to minimize differences in activity due to brain state, although some difference is inevitable.

There is strong agreement in the lowpass filtered data. Both show 600–800 *μ*V_pp_ periodic oscillations and smaller oscillations in the 20–100 Hz range, which is characteristic of LFP activity under ketamine [13]. For spike filtered data, both recording systems show periods of low activity and high activity (bursts of threshold crossing events) at approximately 5–7 Hz, which is also common under ketamine [14]. While the peak-to-peak spike filtered amplitudes from each system are similar, the noise floor of the test chip is visibly higher, which is expected given the additional circuit and aliased electrode noise ( ~5 *μ*Vrms for the test chip and ~2.1 *μV*_*rms*_ for Grapevine in the spike band). LFP waveforms show good agreement between the two systems across all electrodes up to M = 20 (1 slice per channel with 4 discarded). However, for M > 10, the noise of the spike filtered data was substantially higher.

### B. Action Potential Waveform Analysis

**Fig. 4.**
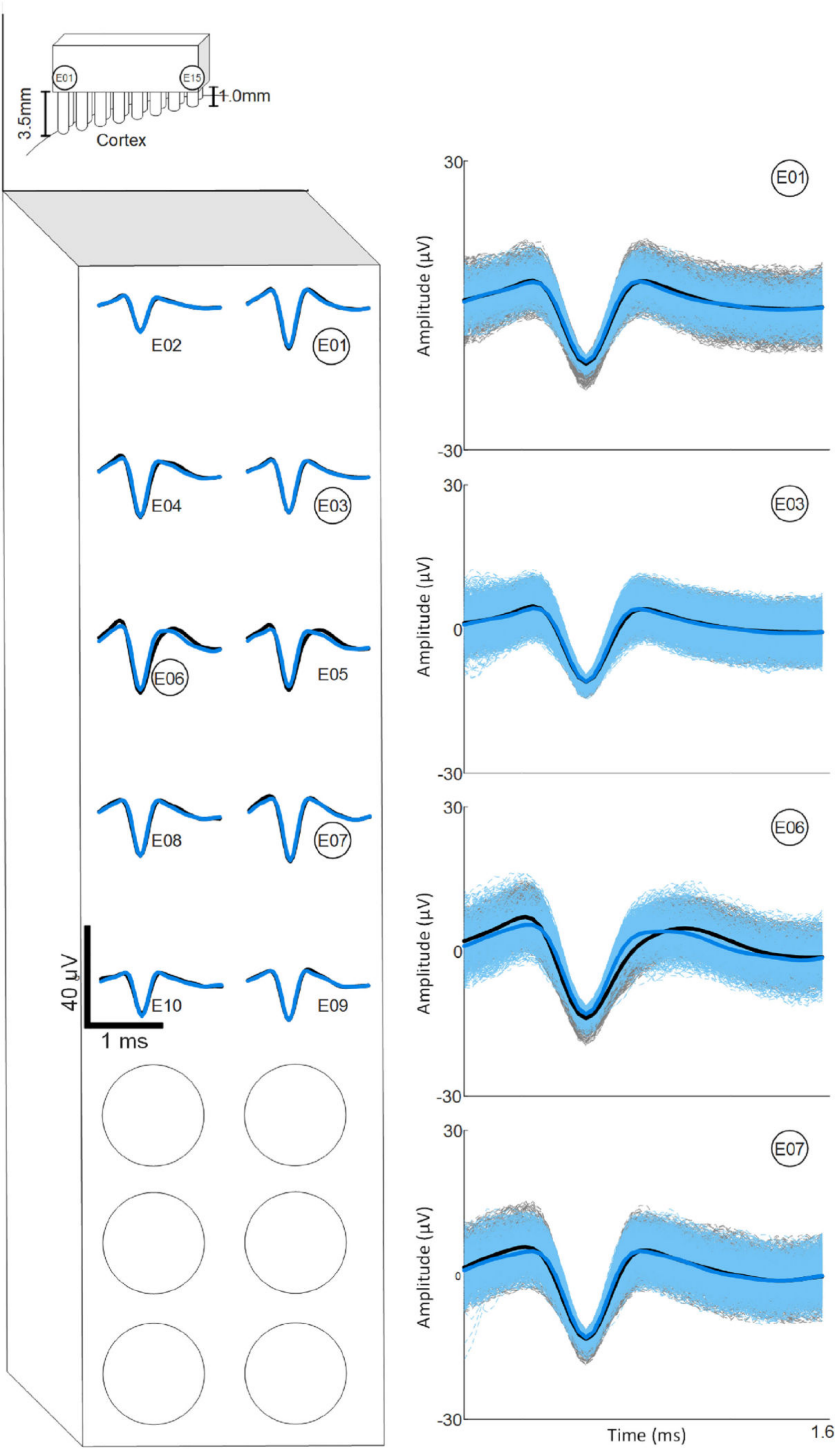
Right: waveforms of threshold crossing events from ~30 seconds of recordings on four example channels. Blue waveforms were recorded from the multiplexed chip; black waveforms from the Grapevine system. Solid traces represent the mean of the shown waveforms; lighter dashed traces show individual threshold crossings. Left: Mean waveforms of threshold crossing events for 10 channels and their approximate location on the electrode array.

To assess the ability for action potential recording, threshold crossing events were isolated and compared to the Grapevine system. Average multiunit waveforms were compared, since there were no clearly discriminable groups of waveforms (putative single units) at any electrode sites when recording with either system. Fig. 4 shows example overlay plots from 4 electrodes. To calculate the average multiunit waveform on each electrode, threshold crossings were averaged across 30 seconds of Grapevine data and 31.5 seconds of test chip data (9 separate 3.5 sec recordings made with M = 10, i.e., 2 slices per channel). Fig. 4 shows close agreement between the mean waveforms of what is likely multiunit activity for Grapevine (black) and the test chip (blue) for the same 10 channels in Fig. 3, at their locations on the array. Similarities in waveform shape between electrodes are likely a result of high neuronal densities in the implanted region and moderate SNR caused by large electrode areas (~4000 *μ*m^2^).

Table II shows quantitative comparisons between the two recording systems. SNR values were calculated using the peak- to-peak amplitude of the average multiunit waveform divided by the twice the standard deviation of noise [15]:

(1) $$SNR=\frac{\max(\bar{W})-\min(\bar{W})}{2*SD_{\varepsilon }}$$

where $\bar{W}$ is the average multiunit waveform and ε is a matrix containing the difference of each sample compared to the average. Noise floors were calculated as *μV*_*rms*_ of the spike-filtered waveforms with threshold crossing time segments removed. As expected, the noise was slightly larger in the test chip recordings but the average SNR and amplitudes were similar (4.23 *μV*_rms_, 2.59 and 15.62 *μV*_*pp*_ respectively compared to 3.17 *μV*_rms_, 3.07 and 16.37 *μV*_*pp*_ for the Grapevine system). Electrode offsets at the test chip input (V_os_,_in_) are listed as well, extracted from the offset correction DAC codes.

**Fig. 5.**
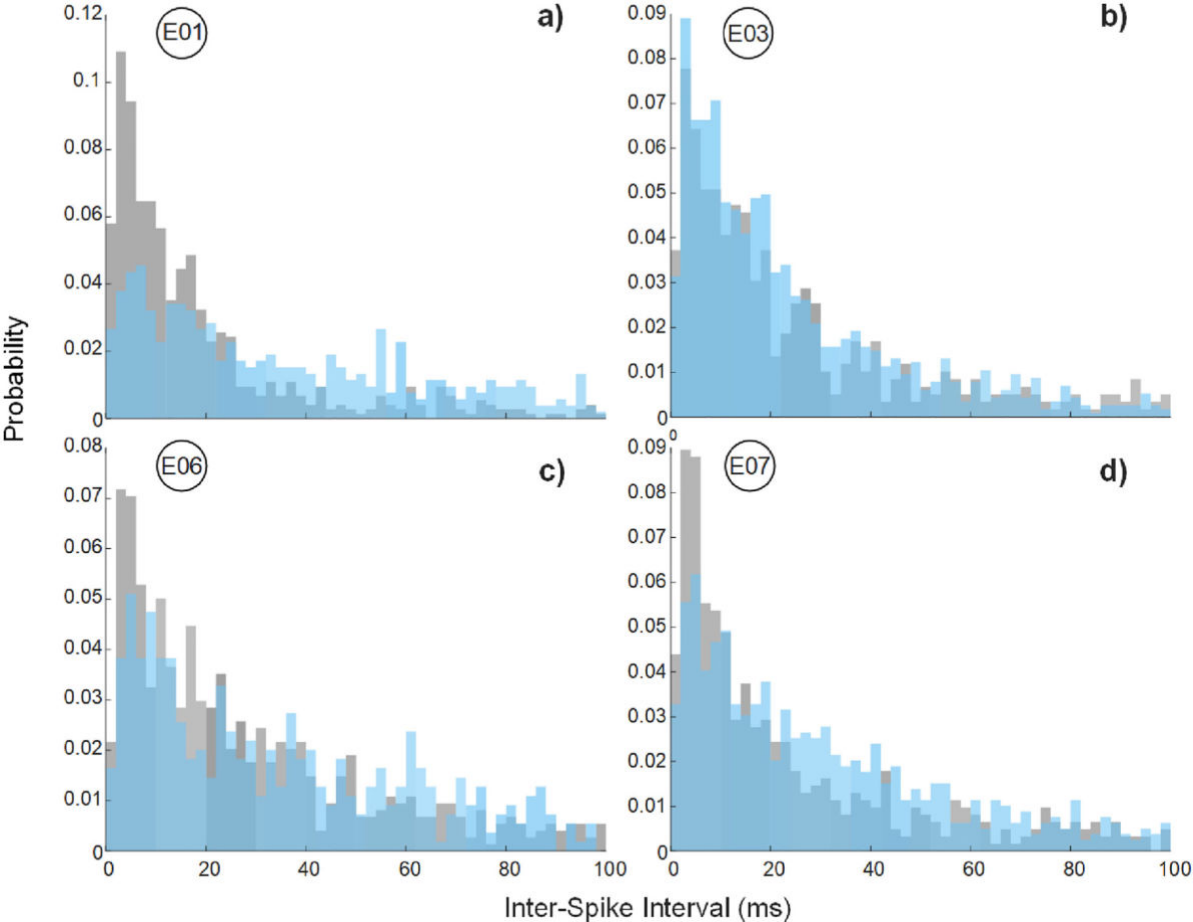
Inter-spike interval (ISI) plots for the four selected channels in Fig. 4 (blue) show close agreement to the Grapevine system (gray).

Fig. 5 shows inter-spike interval (ISI) plots for the same 4 selected channels in Fig. 4. Electrodes 3 and 6 show particularly good agreement. The fact that intervals from all four channels tend toward <20 ms suggests that a majority of them are taking place within the short bursts seen in Fig. 3, which is caused by synchronized firing of nearby neurons.

**Fig. 6.**
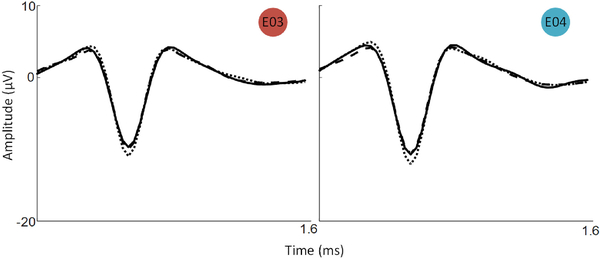
Average waveforms of threshold crossing events for electrodes 3 and 4 at different values of N_av_e. Individual traces show the average waveform for slice allocations of N_ave_ = 20 (solid line), 5 (dashed line) and 2 (dotted line).

Fig. 6 shows the average multiunit waveforms recorded from two channels across a range of N_ave_ values, with each mean waveform derived from 31.5 seconds of recordings taken separately for each value of N_ave_. While there does appear to be a small trend toward increased amplitude and narrowing of the width with decreasing N_ave_, the relative shape is maintained.

### C. Scramble Tests and Non-Uniform Slice Allocation

**Fig. 7.**
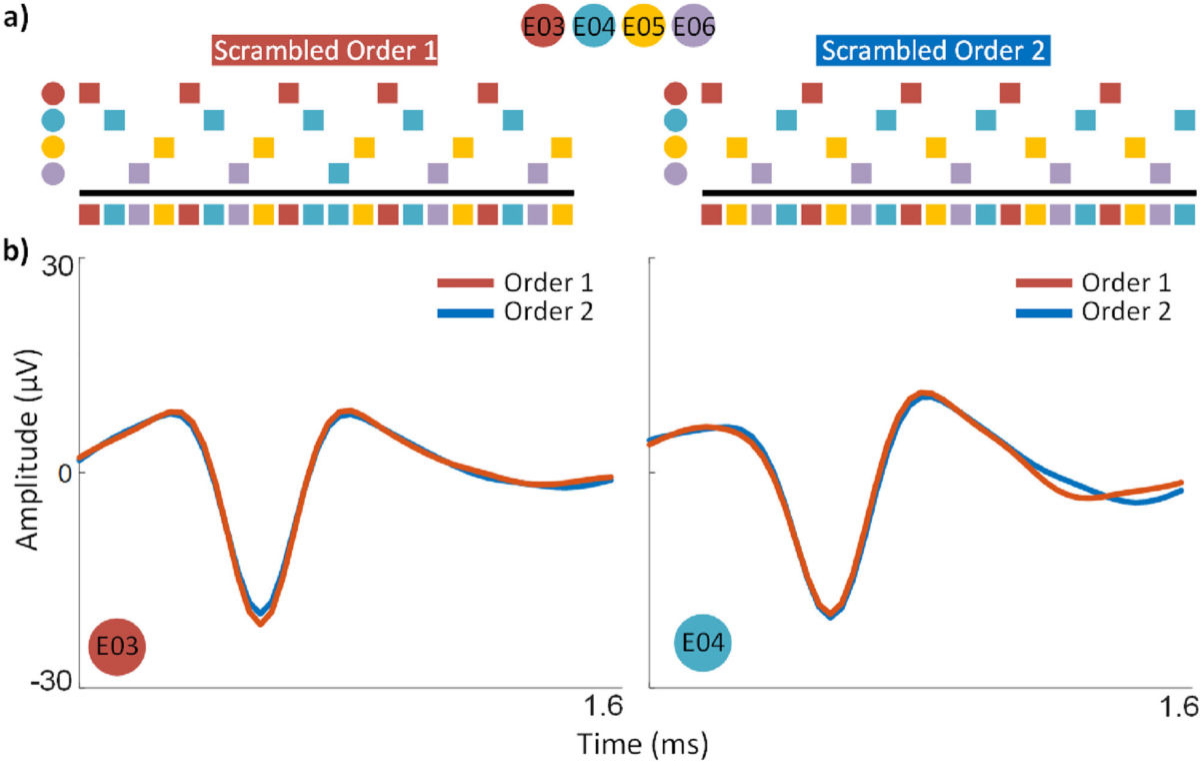
Variations in multiplexing sequence produce little variation in the mean threshold crossing waveform. (a) Diagram illustrating the ordering of the two multiplexing sequences with non-consecutive slices. The region above the black line indicates when each channel was sampled; the region below shows the final sequence. (b) Mean waveforms of threshold crossing events for electrodes 3 and 4 recorded using two different multiplexing orders (1: orange; 2: blue) taken within two minutes of each other.

One potential concern is that a variety of circuit non-idealities and electrode behaviors may result in the multiplexing order having some influence on the shape of the waveforms (e.g., crosstalk or settling). Hence, we compared the average waveforms of four channels with M = 4 for two different orders. Fig. 7 shows the average multiunit waveforms for two example channels. These recordings were taken within two minutes of each other. As Fig. 7 demonstrates, the shapes of the average multiunit waveforms were maintained across the two orders, demonstrating basic confidence that a number of possible issues that can cause artifacts have relatively minor effects at the most.

**Fig. 8.**
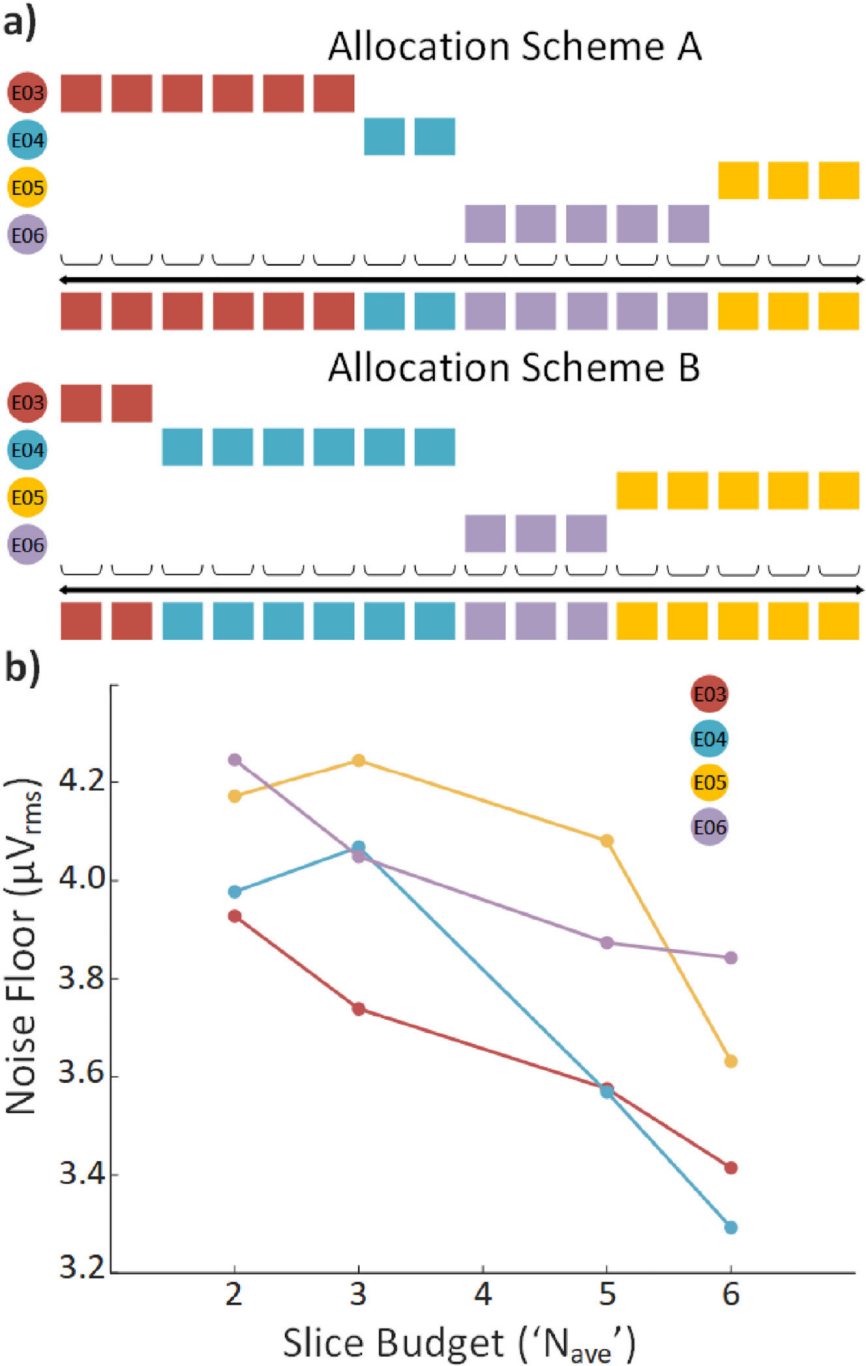
Large slice budget reduces background noise floor. (a) Diagram illustrating the two different slice allocation schemes. (b) Effect of slice budget on background noise floor.

The architecture allows the user to easily assign different slice allocations to individual electrodes to optimize signal quality, e.g., assigning fewer slices to channels with large amplitude action potentials or higher levels of biological noise. Fig. 8(a) demonstrates two example schemes for M = 4 (a 16-slice sequence was repeated and down-sampled to 37.5 kS/s). Electrodes were assigned either 2, 3, 5 or 6 successive slices, which were averaged to reduce noise. Fig. 8(b) shows the background noise floor for all 4 electrodes for four different slice budgets. Table III shows quantitative data for all 4 channels. All recordings show a reduction in noise for a budget of 6 slices versus 2. Thus, this approach enables the ability to digitally optimize noise performance across electrodes, similarly to [16], [17] but using time as a resource instead of large, paralleled circuits that cost high overhead in area.

## V. Noise Analysis

In general, noise in neural recordings primarily comes from three fundamental sources: electronic noise from the circuits, thermal noise from electrode-tissue interface, and background biological activity [18]. To validate the noise performance of the rapidly multiplexed test chip, before the *in vivo* experiments we characterized and recorded from the same microwire electrode array *in vitro* (immersed in phosphate buffered saline solution, PBS). Electrode impedance was measured with EIS to estimate thermal noise using the methods described in [11]. The average electrode impedance at 1 kHz was ~5 kΩ in PBS and ~54 kΩ when implanted, and at 30 kHz was ~2 kΩ in PBS, and ~24 kΩ when implanted (see [6], [11] for representative spectra). We de-embedded the circuit noise from each channel by subtracting a measurement of the test chip’s grounded-input power spectral density (PSD) from the demultiplexed PSD calculated from recordings, similarly to [11]. Here, we compare the de-embedded noise against the impedance-predicted noise, for *in vitro* and *in vivo* experiments. To calculate the predicted thermal noise, the EIS data were converted to a PSD, which was processed with the theoretical WIS transfer function (Fig. 1(d)) and folded around the Nyquist frequency.

Background biological activity that is considered noise in neural recordings is a low frequency phenomenon (up to ~5 kHz [11]). However, circuit noise and the electrode-tissue noise are wideband [11]. As the rapidly multiplexed chip samples the electrodes, high-frequency noise from these sources aliases into the baseband. The PSD of the baseband noise depends on the WIS integration time (set by the multiplexing rate), which determines the front-end’s NEB. As discussed in Section II, for the measurements in this report the multiplexing clock runs at 600 kHz, which translates into 30 kS/s/channel for N_tot_ = 20 and a NEB of 333 kHz (Tint = 1.5 *μ*s).

**Fig. 9.**
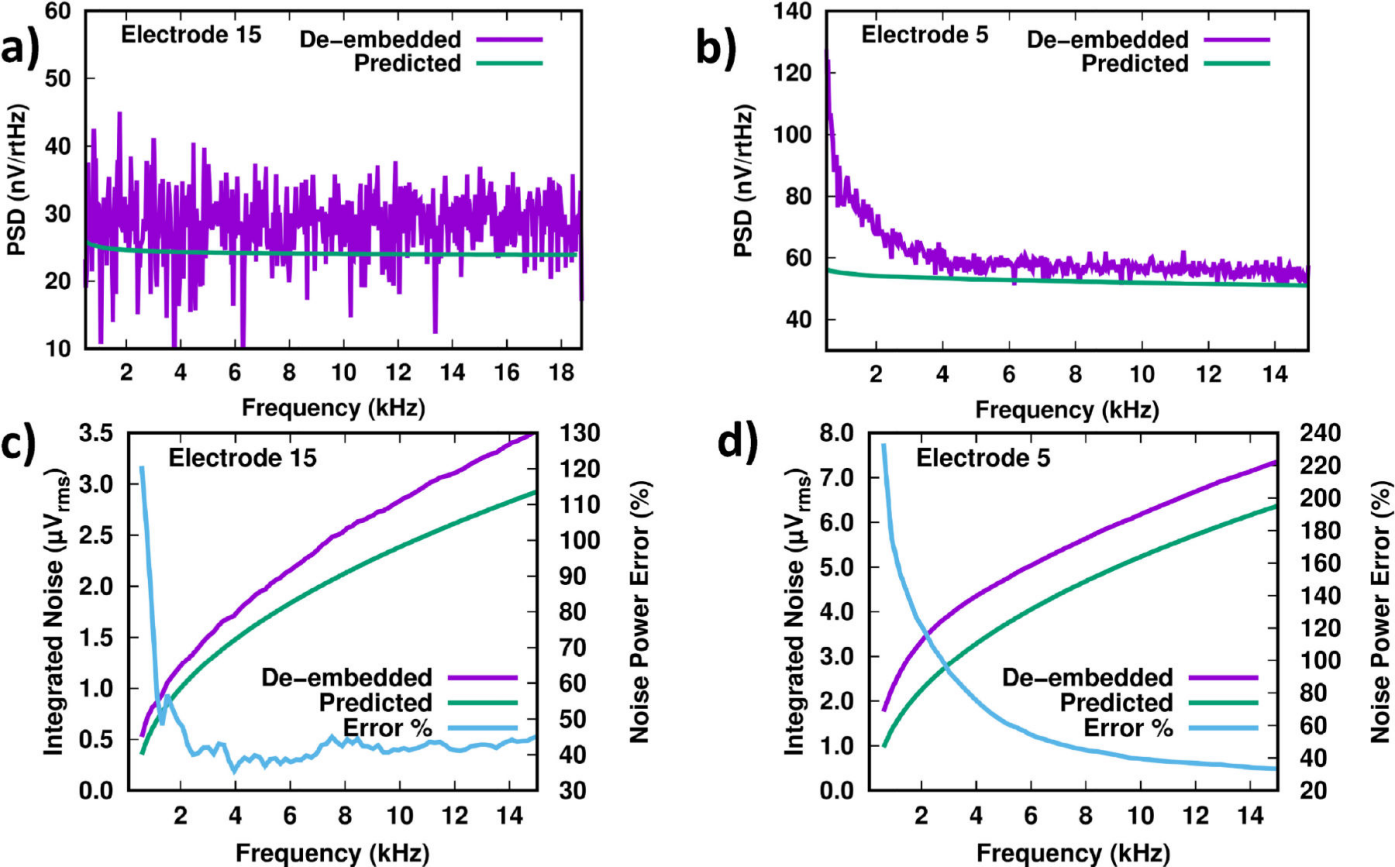
(a) Correspondence between the measured and theoretical baseband noise from a typical electrode (E15) measured *in vitro.* (b) The same analysis for a typical electrode (E05) measured *in vivo,* from the Section IV experiments. (c) A running integral of the measured and theoretical PSDs for the *in vitro* electrode in (a), along with the % error in noise power as a function of the integration frequency. (d) The same analysis for the *in vivo* electrode shown in (b).

We multiplexed all 16 electrodes *in vitro,* using N_ave_ = 1 slice per channel. Fig. 9(a) shows the comparison of the de-embedded PSD for a typical electrode along with the impedance-predicted PSD. Since there is no biological activity, the de-embedded PSD should closely match the predicted thermal noise. We see some error in the matching that is attributed to digital interference in the overall test setup, which we reduced as much as possible through improved shielding and grounding.

Fig. 9(b) shows the same comparison for a typical electrode from the *in vivo* experiments described in Section IV. These measurements were taken under ketamine anesthesia with M = 10 electrodes (N_ave_ = 2 slices per channel), which was the configuration that showed well-isolated threshold crossing activity. We see that the PSD contains low frequency biological activity that causes a deviation with respect to the expected thermal noise, and at higher frequencies the measured PSD is close to the impedance-predicted PSD. Tables IV and V show data for all electrodes tested *in vitro* and *in vivo*, respectively, where the noise was integrated in the 500 Hz-5 kHz band to estimate the noise expected after spike filtering. Somewhat higher errors are seen in the *in vivo* data, which is expected due to the presence of biological activity. The average integrated content in the spike band is 4.77 *μ*V_rms_ for *in vivo* measurements, which correlates well with the background noise seen in the Section IV recordings.

## VI. DISCUSSION

The test chip recordings were strikingly similar to the commercial bench-top system. In general, this is difficult to achieve with custom circuitry due to differences in filter characteristics, linearity, noise, and channel-to-channel variability. In this study, we were able to get agreement by replicating the bench-top system’s analog filters with digital post processing of the test chip data. This match indicates that differences in linearity did not cause significant differences in the shape of the acquired signals (the Grapevine’s linearity is not reported, while the test chip’s THD is 2% [6]). The measured crosstalk depended on the clock duty cycle, since the settling on C_S_ and the reset on CiN,adc happens during the narrow settling phase of the clock. For a 10% duty cycle the measured crosstalk was −20 dB. This is higher than simulated (−60 dB), and is suspected to be due to either parasitic capacitance at the input or higher reset switch resistance compared to simulations. For a 20% duty cycle, the measured crosstalk was < −60 dB. For channel-to-channel variability, we suspect that the Grapevine analog front-end uses G_m_-C filtering, which typically results in high levels of mismatch. The test chip, however, does not have channel-to-channel variability to 1st order since the same circuit is used to condition each channel.

A noise-related aspect that contributed to the close agreement was the reuse of thresholds and “hoops” [12] determined from the bench-top recordings, when processing the test chip data. This step was primarily important for close agreement in the ISI plots, and made a relatively minor impact on the average waveform shape. we did observe some loss of events during strong bursts of activity, when manually isolating threshold crossing events in the test chip data without using knowledge of the bench-top recordings. This loss of events is expected from custom circuitry in general, which is usually designed for higher levels of input-referred noise (5–10 *μ*V_rms_) compared to bulky and power-hungry bench-top systems. This issue is difficult to assess from the literature, due to a lack of studies that perform detailed comparisons of recordings between custom and commercial systems. The isolation method used was deemed appropriate since the main purpose of this study was to verify that rapid multiplexing directly at the electrodes can record the same events as traditional architectures (multiunit action potentials). The experimental results contribute strong evidence that this is the case, although it should be noted that the experimental conditions did not produce recordings of well-isolated single units with either system. In a practical application scenario, the test chip could in fact be used with M = 1 to produce very low noise recordings for initialization of spike sorting, followed by time slice reduction for multichannel acquisition, although this technique is likely not needed in most applications.

In the presented experiments, the multiplexing factor was constrained to 10, primarily due to the small threshold crossing amplitudes (~20 *μ*V_pp_). These amplitudes are on the low side compared to other studies, as are the measured background noise levels (~4 *μ*V_rms_). For example, [19] reported 50–100 *μ*V_pp_ amplitudes and an average background noise level of ~11 *μ*V. A similar longitudinal study [20] reported ^120 jV_pp_ average amplitudes and background noise ranging from 5–13 *μ*V_rms_. Given these amplitudes and noise levels, far higher multiplexing factors are possible, although it should be mentioned that high- frequency noise was not reported and may be somewhat higher given the small electrode areas used. However, [21] reported amplitudes of 60–400 *μ*V_pp_ across multiple types of arrays (including TDT microwires) with different electrode materials and area, with an average SNR of ~6 across and within all types of arrays, with background noise of ~10 *μ*V_rms_. Hence, a multiplexing factor of 10 can be viewed as a lower bound that applies to experiments with very low amplitudes and low intrinsic SNR (~3 in this study). As demonstrated in this report, a system can be designed for higher multiplexing ratios than those that are allowed in one particular experiment, without sacrificing performance, since multiple time slices can be used to reduce noise. The non-uniform slice allocation experiments presented highlight the utility of the rapidly multiplexed approach for designing an adaptive system without large area overhead [16], [17] or the need to perturb the circuit’s bias point.

There are still many fundamental limits and practical issues to be explored, and large open area for refining circuit implementations and performance. while this approach has been recently explored for low impedance surface electrodes for electrocorticography [22], to the authors best knowledge this report and our work in [6] are the only examples in the literature of acquiring putative action potentials using rapid multiplexing, directly at high impedance electrodes, without any preamplification or buffering. Although questions remain related to electrode behavior and fundamental limits (focuses of our ongoing work), this study hopefully contributes confidence in a dramatically different approach to neural recording with tremendous potential for scalability. As discussed in [6], this technique is most beneficial for fully integrated arrays with active circuitry (e.g., [10]), since it does not address the wiring bottleneck that occurs if the circuitry is on a separate chip.

## VII. Conclusion

This report described verification experiments comparing a rapidly multiplexed CMOS test chip to a commercial, bench-top recording system. strong agreement was observed in recordings between the two systems, including threshold crossing shapes and inter-spike intervals. Measurements of noise characteristics were analyzed to verify the fundamental theory of rapid multiplexing using windowed integration sampling, as well as the test chip’s performance. The results indicate successful recording of putative multiunit action potentials using a 10:1 multiplexing factor. This factor divides the circuit’s effective area, resulting in 0.0077 mm^2^/channel, which is >5x smaller than typical multichannel neural recording chips, and reaches the state of the art [10]. Higher multiplexing factors are expected to be achievable, and there is new open space for further research into similar techniques and improved electronics.

## Figures and Tables

**TABLE I:** Noise and Bandwidth of the Commercial System and Test Chip

System	Grapevine	Custom Chip

Input-referred Noise	2.1 μV_rms_	5.5 μV_rms_
Bandwidth	4.9 kHz	15 kHz
High-pass Comer	1 Hz	<0.2 Hz (estimated)

**TABLE II:** Spike-Filtered Analysis and Electrode Offsets

Channel	Pk-Pk Amplitude (μV)	Noise Floor (μV_rms_)	SNR	V_os,in_ (mV)

1	17.32; **16.73**	3.37; **3.92**	2.71; **2.85**	**−20.2**
2	10.50; **10.58**	2.09; **3.28**	3.24; **2.59**	**−4.1**
3	15.68; **15.23**	3.11; **4.39**	2.87; **2.59**	**−10.9**
4	17.87; **16.98**	3.46; **4.41**	3.18; **2.70**	**−13.2**
5	19.03; **16.75**	3.28; **4.47**	3.21; **2.47**	**−10.5**
6	21.07; **18.54**	3.62; **4.47**	3.76; **2.69**	**−11.4**
7	19.15; **17.96**	3.72; **4.79**	2.82; **2.72**	**−13.8**
8	16.32; **16.20**	3.34; **4.65**	2.81; **2.50**	**−13.6**
9	14.73; **14.36**	3.02; **3.47**	2.90; **2.78**	**−11.1**
10	12.06; **12.83**	2.64; **4.43**	3.18; **2.22**	**−13.2**

Light values were calculated from Grapevine system; bold values were calculated from rapidly multiplexed CMOS chip.

**TABLE III:** Variable Slice Allocation

Electrode	Slices	Noise Floor	Pk-Pk Amplitude	*SD*_ε_	SNR

3	2	3.93	15.12	2.90	2.61
	3	3.74	14.95	2.98	2.51
	5	3.58	14.90	2.59	2.88
	6	3.41	14.88	2.78	2.68

4	2	4.00	17.11	3.19	2.68
	3	4.07	17.06	3.25	2.63
	5	3.57	17.24	3.07	2.80
	6	3.3	16.82	2.82	2.99

5	2	4.17	16.84	3.26	2.59
	3	4.25	17.06	3.15	2.71
	5	4.08	16.17	3.27	2.48
	6	3.63	16.56	3.17	2.61

6	2	4.25	18.62	3.31	2.81
	3	4.05	18.11	3.42	2.65
	5	3.87	18.18	3.15	2.71
	6	3.84	18.86	3.11	3.04

**TABLE IV:** In Vitro Noise Comparison Between Measurements and Theory

Electrode	Dembedded Noise (μV_rms_)	Predicted Noise (μV_rms_)	% Error

1	1.7	1.5	13
2	2	1.6	25
3	2.1	1.6	31
4	1.8	1.5	20
5	2	1.5	33
6	2.1	1.6	31
7	1.7	1.5	13
8	2.2	1.4	57
9	2.2	1.6	37
10	2.1	1.6	31
11	1.7	1.5	13
12	2	1.5	33
13	1.8	1.6	12
14	2	1.5	33
15	1.7	1.6	6
16	1.9	1.6	18

Mean	1.94	1.54	12
Stnd Dev	0.18	0.06	25

**TABLE V:** In Vivo Noise Comparison Between Measurements and Theory

Electrode	Dembedded Noise (μV_rms_)	Predicted Noise (μV_rms_)	% Error

1	5.8	4	45
2	5.2	4	30
3	5	4	25
4	3.5	2.5	40
5	4.2	3.2	31
6	4.6	3.2	43
7	5.1	3.7	37
8	5.5	4	37
9	4	3.8	5
10	4.8	3.5	37

Mean	4.77	3.59	11
Stnd Dev	0.67	0.47	33
